# Exposure to visual perturbations elicits adaptation in step kinematics during gait in those with chronic ankle instability

**DOI:** 10.1186/s12984-025-01705-w

**Published:** 2025-10-29

**Authors:** Serkan Uzlasir, Jason R. Franz, Andrew Shelton, Joshua S. Mohess, Erik A. Wikstrom

**Affiliations:** 1 Faculty of Sports Science, Nevsehir Hacı Bektas Veli University, 50300 Nevsehir, Türkiye; 2 Lampe Joint Department of Biomedical Engineering, University of North Carolina at Chapel Hill and North Carolina State University, Chapel Hill, NC USA; 3 MOTION Science Institute, Department of Exercise & Sport Science, University of North Carolina at Chapel Hill, Chapel Hill, NC USA

**Keywords:** Optical flow, Stroboscopic vision, Dynamic balance

## Abstract

**Background:**

Chronic ankle instability (CAI) is associated with impaired gait adaptability, and investigating time-dependent changes in step kinematics and variability—particularly step-to-step corrections—under visually-disruptive conditions compared to a control setting may offer valuable insights into the sensorimotor deficits underlying this condition.

**Methods:**

This cross-over investigation included nineteen participants (Age: 22±3 years, 5±3 sprains, 7±9 giving-way episodes, 23±6 IdFAI score). Inclusion criteria followed International Ankle Consortium guidelines. Participants completed two 15 min walking trials on an instrumented treadmill at 1.2 m/s surrounded by a speed-matched virtual hallway. Within each 15-minute assessment, participants walked normally without visual perturbations for the first (Pre-test) and last two minutes (Post-test). Between these assessments, participants were exposed to 10 min of either continuous mediolateral optical flow perturbations (OFP) or stroboscopic vision (SV) and a 1-minute active rest period. This window allowed us to capture early (first two minutes of exposure) and late (last two minutes of exposure) adaptation. Means and standard deviations of step widths (SW), step lengths (SL), and their respective variabilities (SWV, SLV) were calculated from consecutive heel positions. For each visual condition, separate repeated measures ANOVA were used.

**Results:**

We observed significant changes over time for both OFP and SV (*p* ≤ 0.036). In early adaptation, for both OFP and SV, SL decreased, while SW and SWV increased (*p* ≤ 0.009). In late adaptation for OFP, SL increased (*p* = 0.012) while SLV decreased (*p* = 0.002). During the same period for SV, SW decreased (*p* = 0.002) while SL (*p* = 0.008) increased. Post-test demonstrated reduced SW (*p* ≤ 0.036) and increased SWV (*p* ≤ 0.022) relative to Pre-test for OFP and SV.

**Conclusions:**

This study identified time-dependent changes in step kinematics and variability in response to OFP and SV among individuals with CAI. The observed changes indicate a reduction in anticipatory control and an increase in reactive control with prolonged exposure to visual perturbations. A short-term aftereffect of the step-to-step reactive control strategy was also observed.

**Supplementary Information:**

The online version contains supplementary material available at 10.1186/s12984-025-01705-w.

## Introduction

Lateral ankle sprains (LAS) are among the most common musculoskeletal injuries, accounting for 15% of reported injuries in all sports [1]. More than 40% of individuals who sustain a LAS develop chronic ankle instability (CAI) [2], which is characterized by repetitive episodes of the ankle giving way, recurrent ankle sprains and ongoing symptoms such as pain, weakness and sensorimotor dysfunction [3]. One of the most studied aspects of sensorimotor dysfunction is postural control because poor balance is a risk factor for index and recurrent LAS [4]. Relative to uninjured controls, individuals with CAI have impaired static and dynamic postural control [5, 6] which is hypothesized to be caused, at least in part, by altered sensory organization strategies [7]. 

Sensory organization strategies dynamically shift/reweight based on the fidelity of sensory sources [8]. This ability appears diminished in individuals with CAI [9, 10]. More specifically, those with CAI appear to have an increased reliance on the visual system while maintaining postural control during static stance [11] and while walking [12], likely due to somatosensory damage during the LAS event(s). During dynamic activities of daily life and sport, when vision is required to track objects within the environment [13], a reliance on the visual system to help maintain postural control may partially explain the increased risk of recurrent LAS in those with CAI. Therefore, identifying potential therapeutic interventions that could help to restore appropriate sensory organization strategies is warranted.

Walking balance is achieved, in part, by step-to-step control of foot placement (i.e. step width and step length) [12, 14–17]. The motor planning and execution of foot placement, particularly during balance challenges, is dependent on having appropriate sensory organization strategies [18, 19]. Previously, older adults have exhibited time-dependent changes in walking balance control in response to prolonged optical flow perturbations (OFP) [20]. Initially, older adults responded, at least during early exposure, to unpredictable balance challenges with a cautious anticipatory balance control strategy (i.e. shorter and wider steps) to increase stability and minimize reliance on reactive adjustments [20]. However, with continued exposure the older adults appeared to adopt a more reactive strategy of step-to-step adjustments. After the perturbations stopped, the older adults demonstrated a short-term aftereffect, suggestive of an increase in reactive control and/or an increase in balance confidence [20]. 

Those with CAI, like older adults, demonstrate sensorimotor dysfunction [21] and significant alterations in dynamic balance control, relative to young healthy adults, upon exposure to OFP [12, 18, 22]. Thus, prolonged exposure to OFP may represent a possible approach to restore appropriate sensory organization strategies in those with CAI. Similarly, stroboscopic vision (SV), which is the transient and repeated occlusion of visual information, has been shown to disrupt static postural control in those with CAI [23, 24]. Additionally, SV can enhance sensorimotor adaptation and motor learning during dynamic tasks such as agility drills, or dynamic balance [25–27]. These findings suggest that SV may facilitate sensory reweighting under dynamic conditions by challenging the visual system and promoting greater reliance on proprioceptive and vestibular inputs. Therefore, prolonged use of SV may also help to restore appropriate sensory organization strategies in those with CAI. However, no investigation has quantified the effect of a prolonged bout of OFP or SV on dynamic balance control in those with CAI.

Therefore, the purpose of this study was to quantify the time-dependent changes in step kinematics and variability (i.e., step-to-step adjustments) during exposure to OFP and SV relative to a control condition in those with CAI. We hypothesized that those with CAI would make time-dependent changes in step kinematics as evidenced by shifts away from generalized anticipatory control strategy with prolonged exposure to both OFP and SV. We also hypothesized that those with CAI would exhibit aftereffects in step kinematics following cessation of visual perturbations indicative of increased reactive balance control.

## Methods

### Participants

An a priori power analysis, based on time dependent changes in older adults due to OFP, was completed. We purposefully chose a more conservative effect (0.3) relative to those reported given that the population was younger and because the effect of SV was unknown. Based on the effect size, an alpha level of 0.05, and a power of 0.80, 18 total participants were needed. Therefore, nineteen CAI individuals volunteered for and completed this investigation (Table 1). Those with CAI were required to (1) have sustained at least two lateral ankle sprains; (2) have experienced at least one episode of giving way within the past 6-months; (3) answer “yes” to 4 or more questions on the Ankle Instability Instrument (AII); and (5) have a score > 11 on the Identification of Functional Ankle Instability scale (IdFAI). These criteria align with the guidelines established by the International Ankle Consortium’s recommendations [28]. Seven of the participants had bilateral CAI. Exclusion criteria included being younger than 18 years of age, being older than 35 years of age, having known vestibular and vision problems, acute lower extremity or head injuries (< 6 weeks), chronic musculoskeletal conditions known to affect balance (e.g., ACL deficiency), a history of ankle surgeries to fix internal derangements, and other conditions that may impact balance. Sensitivity to strobe lighting, being knowingly pregnant, having diabetes, and vertigo were also exclusion criteria. All participants read and signed the university-approved informed consent document prior to participation.

**Table 1:** Participant demographics, injury history characteristics and self-reported function

Variable	Mean ± Standard Deviation
Age (years)	22±3
Height (m)	1.7±0.1
Weight (kg)	73.0±12.7
Giving way episodes within past 6 months	7±9
Total ankle sprains	5±3
Identification of Functional Ankle Instability	23±6
Foot & Ankle Ability Measure Activities of Daily Living subscale (%)	88±12
Foot & Ankle Ability Measure Sport Subscale (%)	78±21

### Procedures

**Fig. 1:**
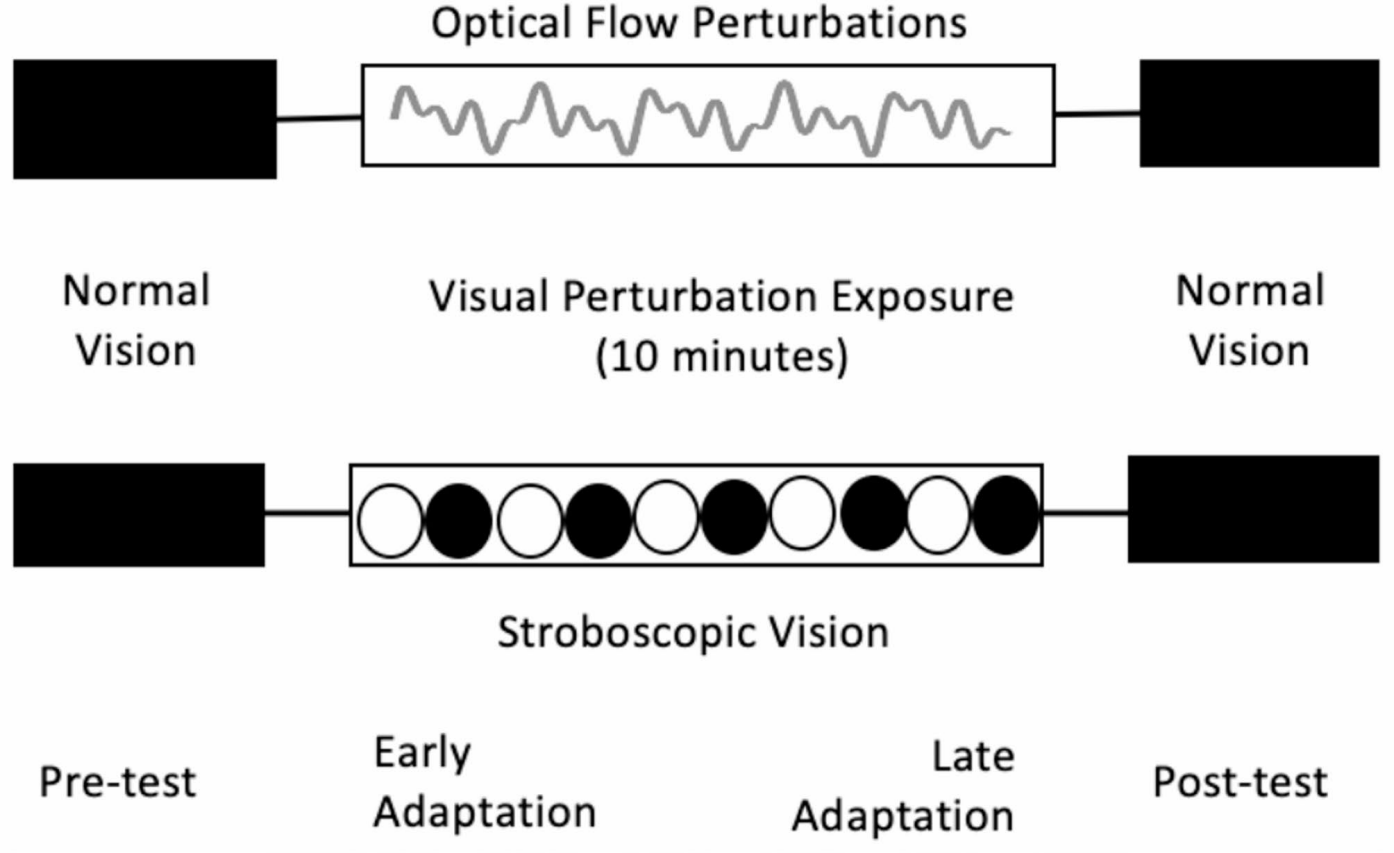
Study protocol illustrating the four assessment periods for both the visually disruptive conditions: optical flow perturbation and stroboscopic goggles

Seventeen retroreflective anatomical markers and an additional 26 tracking markers using rigid clusters were affixed on the 1st metatarsal head, 5th metatarsal head, lateral malleolus, medial malleolus, heel, shank, medial femoral condyle, lateral femoral condyle, thigh, acromion process, anterior superior iliac spine (ASIS), and posterior superior iliac spine (PSIS) bilaterally. Markers were also placed at the C7 vertebra, T10 vertebra, left scapula, manubrium of the sternum, and xiphoid process of the sternum. Once prepped, participants completed two 15-minute gait protocols in a counterbalanced order after the order was randomized for the first participant. Between the protocols, participants were seated for a 15-minute wash out period. During this rest period, participants completed the Foot and Ankle Ability Measure (FAAM) Activities of Daily Living and Sports subscales to quantify functional limitations [29]. For both 15-min trials, participants walked at 1.2 m/s on a split-belt instrumented treadmill (Bertec, Columbus, OH) facing a 2.75 m high and 2.25 m wide semi-circular rear-projection screen [12, 22]. A virtual hallway which mimicked optical flow while walking was set to the same speed as the treadmill belt (Fig. 1).

Within each 15-minute assessment, participants walked normally without visual perturbations for the first 2 min. The next 10-minutes included exposure to OFP or SV. Participants then completed a 1-minute active rest period where treadmill speed was reduced, and the visual perturbations were turned off. Finally, participants concluded the assessment by walking for 2 min without any visual perturbations to determine the retention of any changes (Fig. 1).

The OFP condition manipulated the virtual hallway that participants viewed while walking. The mediolateral (ML) perturbations consisted of the sum of three sine waves (phase, φ = 0), such that a full 0.35 m amplitude was applied at 0.250 Hz and half that amplitude was applied at 0.125 Hz and 0.442 Hz [20]. These perturbations were implemented as translational shifts of the visual field within the frontal plane and have been used extensively in prior studies with well-documented effects on gait in both young and older adults [20, 22]. To account for the projector screen’s curvature, a custom MATLAB (Mathwork, Inc., Natick, MA) warped the projected image onto a geometrical mesh composed of nodes derived from physical measures of the screen’s height, width, radius of curvature, and distance from the projector [19, 22]. 

SV was delivered via stroboscopic goggles (Senaptec Inc., Beverton, Oregon). These 8-oz goggles resemble sport goggles and were secured to the head with a Velcro strap. The goggles were set to level 3, which means the lenses consistently alternated between opaque (150 ms duration) and translucent (100 ms) [30, 31]. Prior to starting the SV condition, participants were informed about the flashing nature of the goggles and were allowed to wear them for a standardized 2-minute accommodation period. These goggles were worn throughout the 15-minute SV protocol but were only active during the 10-minute exposure period. The virtual hallway with optical flow but without OFP was active during the SV condition to give participants an active visual field that could be occluded.

### Data analysis

Kinematic and kinetic data were captured using a 14-camera motion capture system (Motion Analysis Corp., Santa Rosa, CA) operating at 100 Hz. Primary outcomes were based on foot placement measures derived from marker position data. Marker trajectories were low-pass filtered using a fourth-order Butterworth filter with a 12 Hz cut-off frequency. Then, right and left heel-strikes, based on the anteroposterior heel positions relative to the sacral marker, were identified using previously described methods [32]. We calculated right-left and left-right step width (SW) values from consecutive heel marker positions at the instant of heel-strike [18, 22]. Step length (SL) values were also computed as the relative fore-aft position of successive heel markers at heel-strike plus the treadmill translation over the duration of that step [18, 22]. Next, we calculated the variability of step width (SWV) and step length (SLV) as the standard deviations of SW and SL over all steps taken. Outcomes were extracted within the four phases of each walking protocol for further analysis: the first 2 min without perturbations (Pre-test), the first two minutes of perturbation exposure (Early Adaptation), the final 2 min of perturbation exposure (Late Adaptation), and the final 2 min of the walking trial (Post-test).

### Statistical analysis

Data analysis was performed using SPSS software version 22.0 (IBM Corp, Armonk, NY). The normality of the data was assessed using the Shapiro-Wilk test. For OFP and SV, we used separate repeated measures ANOVA with four levels (pre-test, early adaptation period, late adaptation period, post-test) to assess effects relative to unperturbed walking. When appropriate, Bonferroni post-hoc tests were conducted to determine the location of statistically significant differences. All effect sizes were bias corrected Hedge g effect sizes with 95% CIs and were interpreted as < 0.2 = negligible, ≥ 0.2 = small, ≥ 0.5 = moderate, and ≥ 0.8 = large [33]. An a priori alpha level of 0.05 was used to determine statistical significance for all analyses.

## Results

### Optical flow perturbation

**Fig. 2:**
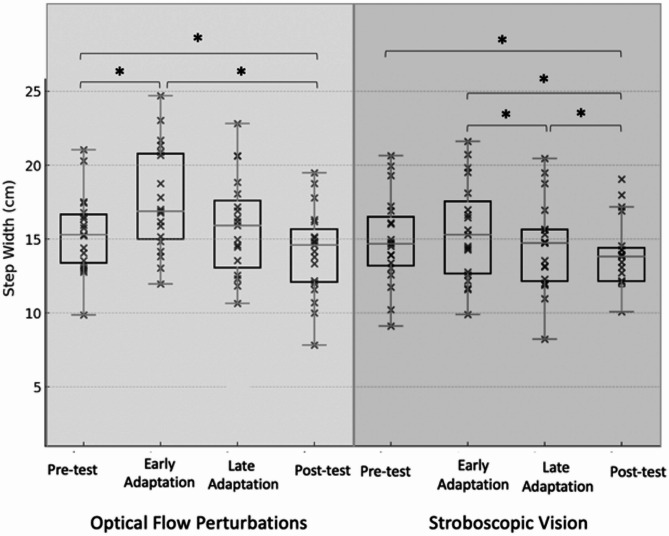
Box-and-whisker plot with individual data points illustrating step width across the Pre-test, Early-adaptation, Late-adaptation, and Post-test assessments for both the optical flow perturbation (left) and stroboscopic goggle (right) conditions. * Indicates statistically significant difference (*p* ≤ 0.05)

**Fig. 3:**
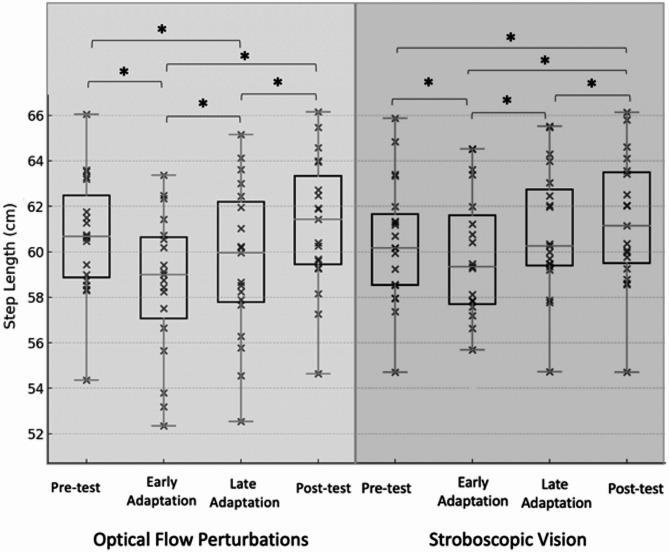
Box-and-whisker plot with individual data points illustrating step length across the Pre-test, Early-adaptation, Late-adaptation, and Post-test assessments for both the optical flow perturbation (left) and stroboscopic goggle (right) conditions. * Indicates statistically significant difference (*p* ≤ 0.05)

**Fig. 4:**
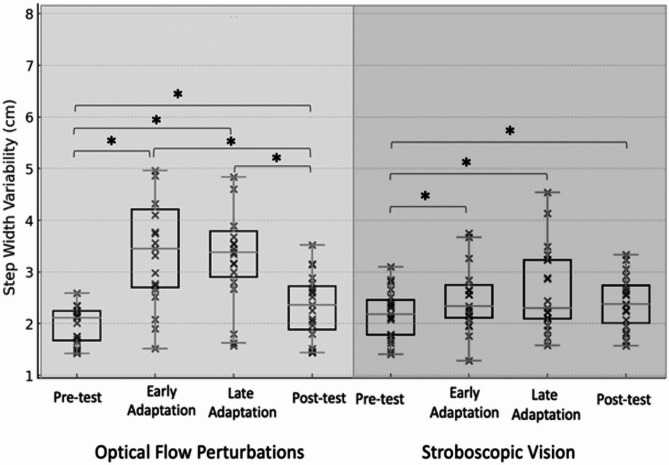
Box-and-whisker plot with individual data points illustrating step width variability across the Pre-test, Early-adaptation, Late-adaptation, and Post-test assessments for both the optical flow perturbation (left) and stroboscopic goggle (right) conditions. * Indicates statistically significant difference (*p* ≤ 0.05)

**Fig. 5:**
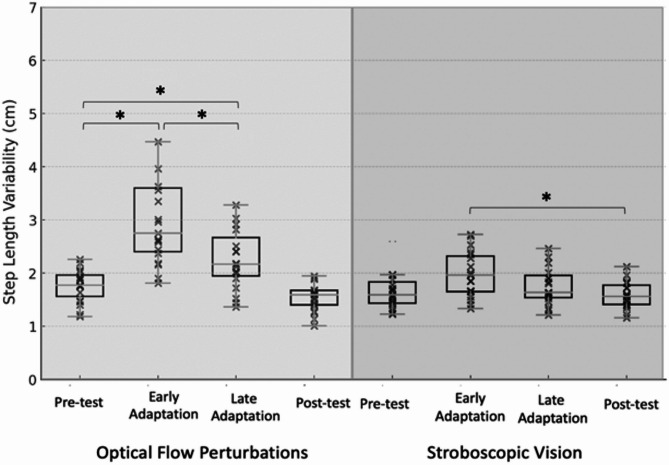
Box-and-whisker plot with individual data points illustrating step length variability across the Pre-test, Early-adaptation, Late-adaptation, and Post-test assessments for both the optical flow perturbation (left) and stroboscopic goggle (right) conditions. * Indicates statistically significant difference (*p* ≤ 0.05)

OFP resulted in significant changes over time for SW, SL, and SWV (*p* ≤ 0.015). Pairwise comparisons illustrated that relative to Pre-test, the early adaptation period elicited decreases in SL (*p* < 0.001) and increases in SW (*p* < 0.001), SWV(*p* < 0.001), and SLV (*p* = 0.002). Thereafter, relative to early adaptation, SL increased (*p* = 0.012) and SLV decreased (*p* = 0.002) for late adaptation. Relative to late adaptation, SL (*p* < 0.001) increased and SWV decreased (*p* = 0.001) for post-test. Post-test demonstrated reduced SW (*p* = 0.036) and increased SWV (*p* = 0.022) relative to Pre-test (Table 2; Figs. 2, 3, 4 and 5). The OFP effect sizes can be seen in Appendix 1.

**Stroboscopic Vision**.

SV resulted in significant changes over time for SW, SL, SWV, and SLV (*p* ≤ 0.036). Relative to Pre-test, the early adaptation period elicited decreased SL (*p* = 0.009) and increased SWV (*p* < 0.001). Thereafter, relative to early adaptation, SL increased (*p* < 0.001) and SW decreased (*p* = 0.004) for late adaptation. Relative to late adaptation, SW (*p* = 0.002) decreased and SL increased (*p* = 0.008) for Post-test. The Post-test, relative to Pre-test, had decreased SW (*p* < 0.001) and increased in SL (*p* < 0.001), and SWV (*p* = 0.05) (Table 2; Figs. 2, 3, 4 and 5). ). The OFP effect sizes can be seen in Appendix 1.

**Table 2:** Means and standard deviations of the step width and length variables across the four assessment time points of the optical flow perturbation condition

	Pre-Test	Early Adaptation period	Late Adaptation period	Post-Test
**Optical Flow Perturbation**				
Step Width (cm)	15.3 ± 2.67	17.6 ± 3.58*	15.2 ± 4.53	14.1 ± 2.98*,^**†**^
Step Length (cm)	60.7 ± 2.63	58.5 ± 3.13*	59.5 ± 3.39*,^**†**^	61.2 ± 2.94^**†**^,^**‡**^
Step Width Variability (cm)	2.01 ± 0.46	3.67 ± 0.15*	3.65 ± 0.16*	2.33 ± 0.62*,^**†**^,^**‡**^
Step Length Variability (cm)	1.76 ± 0.30	3.22 ± 1.34*	2.33 ± 0.75*,^**†**^	3.55 ± 0.88
**Stroboscopic Vision**				
Step Width (cm)	14.8 ± 3.06	15.5 ± 3.35	14.4 ± 3.05^**†**^	13.6 ± 2.73*,^**†**^,^**‡**^
Step Length (cm)	60.4 ± 2.73	59.5 ± 3.31*	60.8 ± 2.80^**†**^	61.3 ± 2.88*,^**†**^,^**‡**^
Step Width Variability (cm)	2.18 ± 0.48	2.55 ± 0.75*	2.64 ± 0.82*	2.41 ± 0.52*
Step Length Variability (cm)	1.65 ± 0.34	1.97 ± 0.42	1.79 ± 0.41	1.58 ± 0.25^**†**^

*Significantly different from Pre-Test (*p* < 0.05), †Significantly different from the Early Adaptation period (*p* < 0.05), ‡Significantly different from the Late Adaptation period (*p* < 0.05)

## Discussion

This study aimed to elucidate the effects of two visually disruptive perturbation methods, OFP and SV, on walking balance control in individuals with CAI. The findings of the current study supported both proposed hypotheses. First, individuals with CAI demonstrated time-dependent changes in step kinematics during prolonged exposure to both OFP and SV. Additionally, those with CAI they exhibited aftereffects in step kinematics following the cessation of visual perturbations, indicating a shift from anticipatory to reactive balance control. Further, the results are consistent with previous investigations demonstrating that the manipulation of visual information alters walking balance control [11, 12] and that time-dependent tuning is possible with prolonged exposure [20]. 

Following the initial exposure to OFP, the adoption of a generalized anticipatory control strategy (i.e. wider and shorter steps) was observed. This adaptation represents a reasonable response to an unknown balance challenges as the response is designed to broaden the base of support and prioritize the maintenance of postural control [20]. However, those with CAI also exhibited greater SWV in the early adaptation period compared to unperturbed walking. Higher foot placement variability suggests that the generalized anticipatory balance control strategy did not meet the participant’s needs as step-to-step adjustments were still required to maintain balance control. This early adaptation to OFP is consistent with changes in step kinematics and variability observed under an identical OFP protocol previously described in uninjured younger [18] and older adults [18, 20]. There, increases in SLV and SWV along with shorter and wider steps were observed upon initiation of the OFP [18]. Song et al. [12] noted the same responses immediately following the initiation of OFP exposure in both those with and without CAI, but the magnitude of step kinematic changes were significantly larger in those with CAI [12].

It is difficult to contextualize our SV results as no other investigation has quantified time-dependent changes in step kinematics following exposure to this visually disruptive modality. Similar to OFP exposure, SV appeared to also elicit a generalized anticipatory strategy. More specifically, shorter and wider steps were observed but the SW change was not statistically significant (*p* = 0.190). Consistent with the OFP results, an increase in SWV variability was also observed in the early adaptation period highlighting that the adopted anticipatory control strategy was not adequate to maintain balance control. Cumulatively, the OFP and SV results reinforce the growing body of evidence suggesting that those with CAI have a disproportionate change in both static and dynamic balance control when visual information is removed and/or disrupted relative to their age matched uninjured control counterparts [11, 12]. 

During the late adaptation phase of the OFP and SV conditions, individuals with CAI exhibited time-dependent changes indicative of a balance control strategy change. More specifically, SW returned to values observed during normal, unperturbed walking during both visual perturbation conditions. SL shifted towards normal walking values with OFP and reached normal walking values with SV. These changes are consistent with patterns observed in both uninjured younger [34] and older adults [20]. It is possible that the CAI participants downweighted the low fidelity visual feedback to ‘ignore’ visual perturbations. However, step-to-step variability remained increased during the late adaptation period relative to normal walking. Thus, despite the sensorimotor dysfunction common in those with CAI, this sample responded to prolonged visual perturbation exposures by shifting away from a generalized anticipatory control strategy in favor of a task-specific reactive control strategy like other populations. This reactive control strategy generates corrective adjustments as challenges to walking balance control are perceived one a step-to-step basis. The adoption of a reactive balance control strategy may also be the result of a multi-sensory reweighting away from visual feedback [35] but this is speculative as sensory organization strategies were not directly measured in this investigation. Regardless of the underlying mechanism, the observed time-dependent effects (i.e. shift toward adoption of step-to-step reactive balance control) should be interpreted as favorable. Previously, the postural control system has been shown to make context-specific neuromuscular adaptations (e.g. decreased intermuscular coupling and bilateral intralimb coherence) in response to 3-minutes of sensory perturbations on the sensory environment [36]. Additionally, a four-session visual perturbation neuromuscular training protocol improved jump landing mechanics and neural efficiency [37]. Both the current and previous results suggest that prolonged exposure to sensory perturbations promote task and environmental specific responses to balance challenges which may facilitate downstream reductions in recurrent injury risk.

Following cessation of exposure to both OFP and SV, participants exhibited longer and narrower steps as well as greater SWV relative to Pre-test. These results may be considered favorable as the data indicates the continued use of a step-to-step reactive balance control strategy after the cessation of prolonged visual perturbations. While speculative, we hypothesize that these short-term aftereffects highlight a reason to leverage repeated bouts of exposure to visually disruptive modalities as a training program for those with CAI. Such a training strategy may facilitate longer-term changes in walking balance control or improvements in resilience to visually disruptive information like what occurs in daily life and sporting events. However, further research with multiple sessions of prolonged exposure to visually disruptive modalities is needed. More importantly, providing prolonged exposure to visual perturbations during less constrained scenarios that better represent tactical and/or sport specific situations (e.g. navigating uneven terrain [38] or dynamic collision avoidance [39] ) are needed to better determine if the results we have observed can be transferred to the real world.

Both visually disruptive perturbations (i.e., OFP and SV) had comparable time-dependent effects on walking balance control. While not analytically assessed, a visual analysis of the data suggests that the OFP had a larger effect on frontal plane parameters (i.e. SW and SWV) particularly during the early adaptation period. We speculate that this is due to the ML perturbation direction and variability of the perturbation magnitude. Given the cost and equipment needed for each of the visually disruptive perturbations used in this investigation, SV - if delivered via goggles - may be the more clinically feasible modality. Incorporating SV into traditional balance training exercises has resulted in greater postural control gains and alterations in sensory organization strategies in those with CAI [25] supporting the premise that it may be a clinically applicable tool.

Our investigation, like all studies, had limitations. First, we only investigated two types of visually disruptive modalities while walking on a treadmill at a fixed speed. Thus, it remains unclear how transferable the results are to real-world conditions. We also only investigated the impact of a brief consistent exposure and cannot speak to the impact, if any, of repeated bouts of exposure to visually disruptive modalities. Finally, seven of the nineteen participants had bilateral CAI. Given that this was a fully within subjects design, we do not believe the presence of bilateral CAI participants would have altered the results.

## Conclusion

Those with CAI demonstrate time-dependent changes in step kinematics and variability as well as short-term aftereffects to prolonged exposure to two visually disruptive modalities: OFP and SV. More specifically, those with CAI appeared to shift toward adopting a step-to-step reactive balance control strategy during and immediately after prolonged exposure to both OFP and SV based on SW and SL returning towards or to baseline values but maintaining continued increases in SWV after the early adaptation period. These results suggest that prolonged exposure to visual perturbations might serve as a possible training strategy to help improve walking balance control in those with CAI.

## Electronic supplementary material

Below is the link to the electronic supplementary material.

Supplementary Material 1

Supplementary Material 2

## Data Availability

Data is provided within the manuscript or supplementary information files. (Additional File 2)
