# Supplementary material for: Exposure to visual perturbations elicits adaptation in step kinematics during gait in those with chronic ankle instability
Source: J Neuroeng Rehabil. 2025 Oct 29;22:229. doi: 10.1186/s12984-025-01705-w (PMC12573960; doi:10.1186/s12984-025-01705-w)
Supplement: Supplementary file 1 — Supplementary Material 1 [file 12984_2025_1705_MOESM1_ESM.docx]

| **Appendix 1:**  Effect sizes and 05% confidence intervals for the changes in the dependent variables during the Optical Flow Perturbation and Stroboscopic Vision protocols. | | | | |
| --- | --- | --- | --- | --- |
|  | **Step Width** | **Step Length** | **Step Width Variability** | **Step Length Variability** |
| **Optical Flow Perturbation** |  |  |  |  |
| Pre-Test---EAP | **0.86 (0.82 to 2.27)** | **-0.84 (-2.18 to -0.75)** | **3.61 (3.73 to 6.32)** | **4.87 (0.24 to 1.57)** |
| Pre-Test---LAP | -0.04 (-0.65 to 0.62) | **-0.46 (-1.56 to -0.23)** | **3.57 (3.04 to 5.31)** | 1.90 (-0.02 to 1.28) |
| Pre-Test---Post-Test | -0.45 (-1.29 to 0.01) | 0.19 (-0.13 to 1.16) | **0.7 (0.11 to 1.42)** | **5.97 (1.43 to 3.06)** |
| EAP---LAP | -0.67 (-1.09 to 0.2) | **0.32 (0.08 to 1.40)** | -0.13 (-0.88 to 0.40) | **-0.66 (-1.40 to -0.08)** |
| EAP---Post-Test | **-0.98 (-1.98 to -0.58)** | **0.86 (0.80 to 2.24)** | **-8.93 (-4.77 to -2.67)** | 0.25 (-0.38 to 0.90) |
| LAP---Post-Test | -0.24 (-0.84 to 0.43) | **0.50 (0.81 to 2.25)** | **-8.25 (-4.61 to -2.56)** | **1.63 (0.64 to 2.05)** |
| **Stroboscopic Vision** |  |  |  |  |
| Pre-Test---EAP | **0.70 (0.89 to 2.35)** | **-0.33 (-1.51 to -0.18)** | **0.77 (0.08 to 1.39)** | **0.94 (0.56 to 1.96)** |
| Pre-Test---LAP | **0.29 (0.01 to 1.32)** | 0.15 (-0.23 to 1.06) | **0.96 (0.12 to 1.44)** | 0.41 (-0.36 to 0.92) |
| Pre-Test---Post-Test | 0 (-0.64 to 0.64) | **0.33 (0.35 to 1.7)** | 0.48 (-0.03 to 1.28) | -0.21 (-0.85 to 0.43) |
| EAP---LAP | **-0.33 (-1.49 to -0.17)** | **0.39 (0.42 to 1.79)** | 0.12 (-0.52 to 0.75) | -0.43 (-1.03 to 0.25) |
| EAP---Post-Test | **-0.57 (-2.22 to -0.78)** | **0.54 (0.92 to 2.39)** | -0.19 (-0.86 to 0.41) | **-0.93 (-1.69 to -0.34)** |
| LAP---Post-Test | **-0.26 (-1.69 to -0.34)** | **0.18 (0.13 to 1.48)** | -0.28 (-1.24 to 0.06) | -0.51 (-1.21 to 0.09) |

Early Adaptation Period: EAP. Late Adaptation Period: LAP
